# Recognizing asymptomatic bacteriuria in the surveillance of catheter-associated urinary tract infections—beyond fever and positive urine culture

**DOI:** 10.1017/ash.2024.459

**Published:** 2024-11-14

**Authors:** Hayato Mitaka, Chloe Bryson-Cahn, Jorge O. Estebane, Vanessa A. Makarewicz, John B. Lynch, Jeannie D. Chan

**Affiliations:** 1 Division of Allergy & Infectious Diseases, Department of Medicine, University of Washington School of Medicine, Seattle, WA, USA; 2 Infection Prevention and Control, Harborview Medical Center, Seattle, WA, USA; 3 Department of Pharmacy, Harborview Medical Center, and University of Washington School of Pharmacy, Seattle, WA, USA

## Abstract

Among 143 cases of National Healthcare Safety Network (NHSN) catheter-associated urinary tract infections (CAUTI), 40% were considered catheter-associated asymptomatic bacteriuria (CA-ASB), and 18% clinical CAUTI. An alternative source of fever was present in 70% of CA-ASB. NHSN CAUTI may not be an effective metric for tracking hospital-level infection prevention efforts.

## Introduction

Catheter-associated urinary tract infection (CAUTI) is a healthcare-associated infection (HAI), and reporting is required as a quality metric incentivized by Centers for Medicare and Medicaid Services reimbursement. The surveillance definition for CAUTI includes fever with or without localized urinary symptoms in the presence of indwelling urinary catheter and positive urine culture as defined by the CDC’s National Healthcare Safety Network (NHSN). Although this definition is intended to be objective, accurate, and reproducible,^1^ the reliance on fever and positive urine culture likely overcalls “true” infections attributed to CAUTI. Catheter-associated asymptomatic bacteriuria (CA-ASB), presence of bacteria due to colonization of the urinary tract or urinary catheter, is ubiquitous and the incidence reaches 100% with a month of catheterization.^2,3^ Patients dependent on long-term catheters with CA-ASB easily meet the NHSN CAUTI definition, which is susceptible to any hospital-acquired fever and urine culture practices,^4^ resulting in mandatory HAI reporting. Consequently, the NHSN surveillance definition has been called into question as a performance and quality metric.^4–6^ Although many interventions have been implemented to reduce NHSN CAUTI, a large portion of the reportable events are likely CA-ASB.^7,8^ However, the prevalence of this phenomenon is not well described. We aim to quantify the prevalence of CA-ASB among NHSN CAUTI cases.

## Methods

We performed retrospective chart review of patients ≥18 years old with NHSN CAUTI between July 2022 and July 2023 at Harborview Medical Center, a 500-bed acute care public teaching hospital, and level-1 trauma and burn center. Patients who met the NHSN CAUTI criteria adjudicated by the infection prevention team, performed as part of hospital regulatory requirements, were included. Baseline demographics, clinical and microbiologic data, and antibiotic therapy were abstracted from medical records. Based on prespecified criteria, NHSN CAUTI cases were classified into three categories: clinical CAUTI, CA-ASB, and indeterminate. Patients were considered to have clinical CAUTI if (1) positive blood culture with matching urinary organisms, (2) fever or leukocytosis plus signs and symptoms of UTI such as suprapubic pain, dysuria after catheter removal, or costovertebral angle tenderness, (3) urinary or pelvic symptoms consistent with UTI, or (4) imaging findings supporting UTI. Patients were considered to have CA-ASB if they had (1) a confirmed alternative source of fever, (2) no urinary or pelvic symptoms compatible with UTI, (3) an established alternative etiology of urinary or pelvic symptoms, or (4) absence of pyuria (≤ 5 leukocytes per high-power field) in non-neutropenic patients. All other patients were classified as indeterminate for CAUTI, including those who had (1) fever or leukocytosis with other potential but not confirmed source, and (2) fever or leukocytosis without localizing urinary tract symptoms. Chart review was performed by one infectious diseases physician (HM) with random cases independently reviewed by a second investigator (JBL) to ensure validity. Discordant case classification was reconciled by discussion and consensus. The institutional review board of the University of Washington approved the study and waived written informed consent (IRB ID: STUDY00019574).

## Results

We reviewed 143 patients with NHSN CAUTI; the median age was 58 (IQR: 40–69) with 41% female. Median catheter duration was 9 days (IQR: 5–16). Twenty-six patients (18%) met criteria for clinical CAUTI, while 57 patients (40%) had CA-ASB. The remaining 60 patients (42%) were indeterminate. Approximately 90% of the patients received systemic antibiotics during the NHSN CAUTI event. Among 26 patients meeting clinical CAUTI criteria, 10 (38%) had bacteremia associated with UTI, while 16 (62%) had urinary or pelvic symptoms consistent with UTI. Among 57 CA-ASB patients, 40 (70%) had a confirmed alternative source of fever or leukocytosis, most commonly lower respiratory tract infection (Table 1).

**Table 1.** Patient characteristics and rationale for classification of National Healthcare Safety Network (NHSN) catheter-associated urinary tract infection (CAUTI) cases by clinical chart review

	NHSN CAUTI cases	Treated with antibiotics
Patients with NHSN CAUTI	143	129 (90%)
Median age, year [IQR]	58 [40–69]	
Female sex (%)	59 (41)	
Medical service (%)		
Medicine	24 (17)	
Surgery	23 (16)	
Neurology	18 (13)	
Neurocritical care	17 (12)	
Surgical ICU	15 (10)	
Orthopedic surgery	11 (8)	
Rehabilitation unit	10 (7)	
Medical ICU	8 (6)	
Neurosurgery	7 (5)	
Burn ICU	1 (0.7)	
Other	9 (6)	
Median duration of indwelling urinary catheter, day [IQR]	9 [5–16]	
Clinical catheter-associated urinary tract infection (CAUTI)	26 (18%)	26 (100%)
Bacteremic UTI	10	
Fever or leukocytosis plus symptoms of UTI	8	
Urinary or pelvic symptoms consistent with UTI	8	
Imaging findings supporting UTI	0	
Catheter-associated asymptomatic bacteriuria (CA-ASB)	57 (40%)	46 (81%)
Fever or leukocytosis with confirmed alternative source	40	
Lower respiratory tract infections	18	
Bacteremia	9	
Non-infectious etiologies	3	
Viral infections (eg, Covid-19 and influenza)	3	
*Clostridioides difficile* infection	2	
Intraabdominal infections	2	
Skin and soft tissue infections	2	
Central nervous system infections	1	
No urinary or pelvic symptoms compatible with UTI	9	
Alternative etiologies of urinary or pelvic symptoms	5	
Absence of pyuria in non-neutropenic patients	3	
Indeterminate	60 (42%)	57 (95%)

ICU, intensive care unit; UTI, urinary tract infection; IQR, interquartile range.

## Discussion

We report a high prevalence of CA-ASB ascertained by clinical chart review in patients with NHSN CAUTI at our institution. Despite multiple modifications of the NHSN definition to more accurately reflect clinical CAUTI, including the 2015 update increasing urine culture colony count, our study demonstrated that CA-ASB still represents a large portion of NHSN CAUTI cases due to its reliance on fever and positive urine culture irrespective of the presence of an alternative source of infection. Concern about validity of the current surveillance definition has been echoed by others advocating for reform in CAUTI metrics to better reflect infectious and non-infectious complications of catheter use.^4,5^

Systemic antibiotic treatment was given to 90% of patients at the time of the NHSN CAUTI event, and among CA-ASB cases, 70% had an alternative source of fever. Given a high rate of colonization in catheterized patients, optimization of urine culture testing is necessary because ASB is a key driver of inappropriate antibiotic use, leading to adverse events, emergence of resistance and *Clostridiodies difficile* infection.^9^

Our study has several limitations. The retrospective, single-center experience at a tertiary center specializing in neurological trauma with a high proportion of patients with long-term urinary catheters and lack of restrictions on urine culturing may limit generalizability. Chart review introduces the potential for observer bias, and only a proportion of charts were reviewed by two investigators. However, distinguishing between CA-ASB and CAUTI requires careful clinical judgment and large-scale database studies are not often possible. Case definitions are the cornerstone of national HAI surveillance and should capture true patient harm as accurately as possible to guide hospitals in allocating limited resources. Revisiting quality metrics related to urinary catheter use can help hospitals redirect their efforts and strategies for infection prevention, considering a significant number of CA-ASB cases that are misclassified as CAUTI.
